# Emerging trends and hotspots of the itch research: A bibliometric and visualized analysis

**DOI:** 10.1111/cns.14514

**Published:** 2023-10-30

**Authors:** Jun Li, Liya Wang, Suqing Yin, Shuangshuang Yu, Yanyu Zhou, Xiaoqi Lin, Yingfu Jiao, Weifeng Yu, Xiaoqiong Xia, Liqun Yang, Po Gao

**Affiliations:** ^1^ Department of Anesthesiology, Renji Hospital Shanghai Jiao Tong University School of Medicine Shanghai China; ^2^ Key Laboratory of Anesthesiology (Shanghai Jiao Tong University) Ministry of Education Shanghai China; ^3^ Department of Anesthesiology Chaohu Hospital Affiliated to Anhui Medical University Chaohu Anhui China; ^4^ Department of Gynecologic Oncology, International Peace Maternity and Child Health Hospital, Shanghai Municipal Key Clinical Specialty, Shanghai Key Laboratory of Embryo Original Disease Shanghai Jiao Tong University School of Medicine Shanghai China; ^5^ Department of Dermatology Chaohu Hospital Affiliated to Anhui Medical University Chaohu Anhui China

**Keywords:** bibliometric analysis, inflammation, itch, neuroimmunology

## Abstract

**Aims:**

Itch, a common uncomfortable sensory experience, occurs frequently in inflammatory or allergic disorders. In recent years, with the discovery of itch‐specific pathways in the peripheral and central nervous system, the association between immunology and neural pathways has gradually emerged as the main mechanism of itch. Although many studies have been conducted on itch, no bibliometric analysis study focusing on this topic has been conducted. This study aimed to explore the research hotspots and trends in the itch field from a bibliometric perspective.

**Methods:**

Publications relevant to itch, published from 2003 to 2022, were retrieved from the Science Citation Index‐Expanded of Web of Science Core Collection. Publications were critically reviewed and analyzed with CiteSpace software, Vosviewer, and the bibliometric online analysis platform. Visual maps were conducted in terms of annual production, collaborating countries or institutions, productive authors, core journals, co‐cited references, and keyword bursts.

**Results:**

2395 articles on itch that met our criteria were identified and the quantity of publications has been increasing rapidly since 2012. The USA was the most influential country. University Hospital Münster was the institution with the most publications. Gil Yosipovitch was the most prolific author. Atopic dermatitis (AD), intradermal serotonin, chronic pruritus, mechanical itch, gastrin‐releasing peptide, substance p, interleukin‐31 receptor, histamine‐induced itch, bile acid, scratching behavior, and h‐4 receptor were the top 11 clusters in co‐citation cluster analysis. Keyword burst analysis suggested that treatment, inflammation, and AD are current research hotspots.

**Conclusion:**

Global publications on itch research have increased steadily and rapidly over the past 20 years. Inflammation and AD are current research hotspots. The neuroimmunological and neuroinflammatory mechanisms of itch, as well as clinical assessment methods and therapeutic targets, will be novel research directions in the future. This study provides guidance for further itch research.

## Graphical abstract

This study conducted a comprehensive bibliometric and visual analysis of itch research. 2314 papers closely related to itch research published between 2002 and 2021 were retrieved from the SCI‐E of Web of Science Core Collection database. Full records and cited references were extracted from relevant publications. Then the data were imported into the Online Analysis Platform of Biobliometry, VOSviewer, CiteSpace software, and Bibliometrix to systematically evaluate publication trends in itch research and predict future research directions.

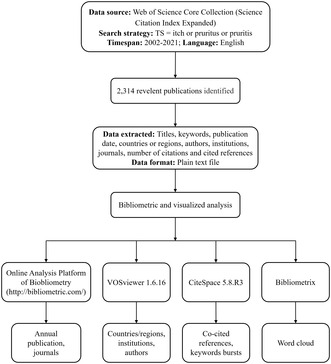

## 1 INTRODUCTION

Itch, or pruritus, was first defined in the 1600s as “an unpleasant sensation that evokes a desire or reflex to scratch.”^1^ The itch sensation is closely associated with a variety of disorders, including inflammatory skin diseases and allergic disorders, such as atopic dermatitis (AD), allergic contact dermatitis (ACD), and chronic urticaria. Acute or chronic pruritus affects approximately 15% of the population and has a significant negative impact on sleep, mental health, and patient's quality of life.^2^ However, in contrast to pain, no Food and Drug Administration‐approved drugs are available for the treatment of itch. Thus, researchers and clinicians must continue exploring the mechanisms of itch to identify possible therapeutic targets.

Itch was once recognized as a mild form of pain. Itch‐specific sensory neural pathways have gradually been revealed with the discovery of gastrin‐releasing peptide (GRP) and its receptor (GRPR).^3^, ^4^ In recent years, the connection between immune and pruritic neural pathways has been gradually revealed with the identification of other itch‐specific mediators such as the Mas‐related G protein‐coupled receptor (Mrgpr) family,^5^, ^6^ natriuretic peptide B (Nppb)^7^ and various itch‐mediating cytokines.^8^ The emergence and ongoing breakthroughs in the field of itch neuroimmunity have revealed that multiple immune cells and secreted cytokines can directly activate sensory neurons to encode itch signals.^9^

The first barrier against external environmental stimulation is the skin, which is innervated by primary sensory neurons. The itch sensory signals originate from skin and sensory nerve endings which respond to inflammatory mediators released by immune cells such as mast cells and T cells, which in turn lead to the depolarization of neuronal cell membranes via the activation of transient receptor potential (TRP) channels TRPV1 and TRPA1 in sensory neurons.^10^ Itch sensory signals can be transmitted from primary sensory neurons to the dorsal horn neurons of the spinal cord, which travel to the cerebral cortex.^11^ A growing number of studies have shown that type 2 immunity is highly relevant to itch. Activated mast cells can release various inflammatory mediators such as histamine, which enables TRPV1 to open and transmit itch signals by activating H1 receptors in sensory neurons.^12^ Skin barrier dysfunction causes epithelial cells to produce the cytokines thymic stromal lymphopoietin (TSLP) and interleukin (IL)‐33. TSLP can trigger itch by binding to TSLP receptors (TSLPR) in sensory neurons.^13^ In a previous study of ACD, IL‐33 was shown to aggravate pruritus caused by skin inflammation through direct stimulation of sensory neurons.^14^ During AD, basophils stimulated by allergens contribute to the exacerbation of AD pruritus through the release of leukotriene C4 that binds to the cysteinyl LT receptor 2 on itch‐associated nonpeptidergic neurons and mediates itch via TPPA1 and TRPV1.^15^

Of the many type 2 cytokines, IL‐31 was the first identified to mediate pruritus by acting directly on sensory neurons.^16^, ^17^ IL‐4 and IL‐13 can also mediate itch by stimulating IL‐4Rα, a receptor on sensory neurons, through the JAK–STAT pathway in lymphocytes.^18^ Beyond cytokines, chemokines can also directly facilitate itch in the sensory nervous system. For example, CXCL10 can directly activate a subpopulation of dorsal root ganglion (DRG) neurons via its receptor CXCR3.^19^ Similarly, CCL2 can induce an itch sensation by directly binding to the receptor CCR2 in sensory neurons.^20^ Neuropeptides released by sensory neurons, such as CGRP (calcitonin gene‐related peptide), SP (substance P), and VIP (vasoactive intestinal peptide), regulate both skin inflammation and itch sensation.^21^, ^22^, ^23^ As such, neuroimmunity is a key mechanism in the development of itch.

Bibliometric analysis is an emerging field of information science that provides a comprehensive review of publication characteristics for a specific publication period.^24^, ^25^ Bibliometric analysis enables qualitative and quantitative assessment of a research field and can help researchers gain insight into current research hotspots and future research trends.^26^ However, to the best of our knowledge, no bibliometric analysis has been published on the topic of itch. Therefore, in this study, we aimed to evaluate the publication trends in itch from 2003 to 2022 to help advance research in this field.

## 2 METHODS

### 2.1 Data sources and search strategies

**FIGURE 1:**
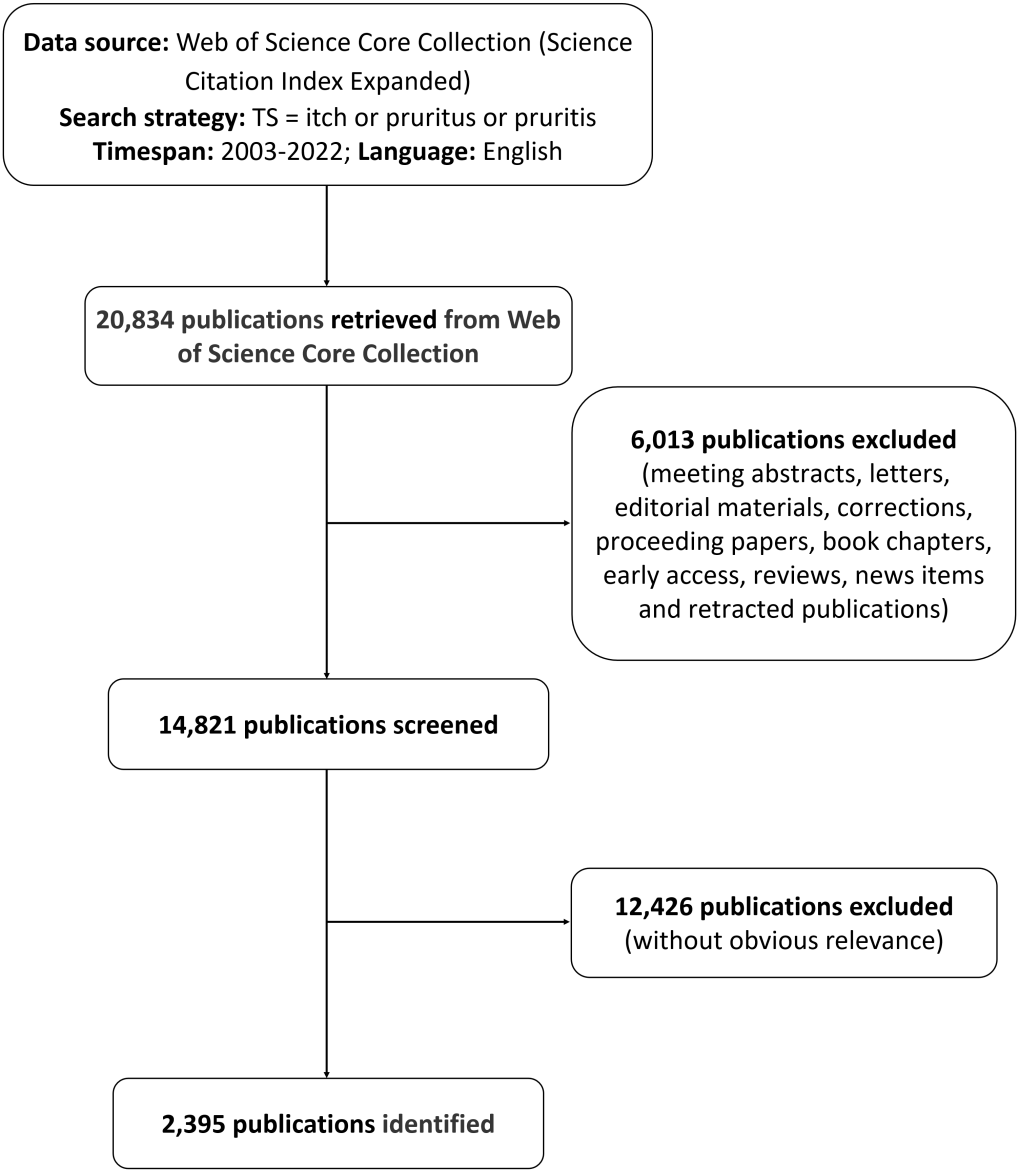
A flowchart for including and excluding publications in itch research.

Based on previous publications, we identified the Web of Science Core Collection (WOSCC) as the most suitable database for bibliometric analysis.^27^ Therefore, we conducted a comprehensive literature search related to itch within the Science Citation Index‐Expanded (SCI‐E) of the WoSCC database from 2003 to 2022. The search and data download of all publications were completed within 1 day to prevent bias caused by frequent database updates. The search terms and strategies used were as follows: TS = (itch or pruritus or pruritis) AND Language = (English), with a limited period set from January 1, 2003 to December 31, 2022. To ensure that the retrieved publications were related to the main topic of this study, two researchers (Jun Li and Liya Wang) screened and recorded all publications by reading the titles and abstracts. The exclusion criteria were as follows: (1) no significant relationship to itch; (2) articles were included but meeting abstracts, letters, reviews, editorial materials, proceedings papers, book chapters, early access, corrections, news items, and retracted publications were not; and (3) duplicate publications. The detailed screening flow chart is shown in Figure 1.

### 2.2 Data collection

After screening the manuscripts, we extracted data from relevant publications, including titles, keywords, publication date, countries or regions, authors, institutions, journals, and number of citations. Full records and cited references of the retrieved articles were downloaded from the WoSCC database. Then the data were converted to a TXT format and imported into the Online Analysis Platform of Biobliometry (http://bibliometric.com/); CiteSpace 5.8.R3 (Drexel University, Philadelphia, USA) and VOSviewer 1.6.16 (Leiden University, Leiden, The Netherlands) for bibliometric analysis.

### 2.3 Bibliometric analysis

We conducted a comprehensive bibliometric analysis of several literature characteristics, including countries/regions, institutions, authors, journals, co‐cited references, and keywords with the strongest citation bursts. The Online Analysis Platform of Biobliometry was used to analyze the number of annual publications and the publication trend in different countries or regions. VOSviewer, developed by Leiden University, is a software tool for constructing visual bibliometric maps.^28^ It can be used to build viewing networks of various countries/regions and institutions based on collaborative data or co‐occurrence data. CiteSpace is a practical visual analysis software, applied to identify new trends and developments in a research field, especially in the analysis of literature citations and keywords.^29^ To identify the current status and hotspots of pruritus research, and to predict future research directions in this field, we used Citespace for co‐citation analysis of reference and keyword burst detection.

All data were published and obtained directly from a public database and no further experiments were performed in this study. Therefore, additional ethical board approvals were not required.

## 3 RESULTS

### 3.1 Quantity and trends analysis of published papers

**FIGURE 2:**
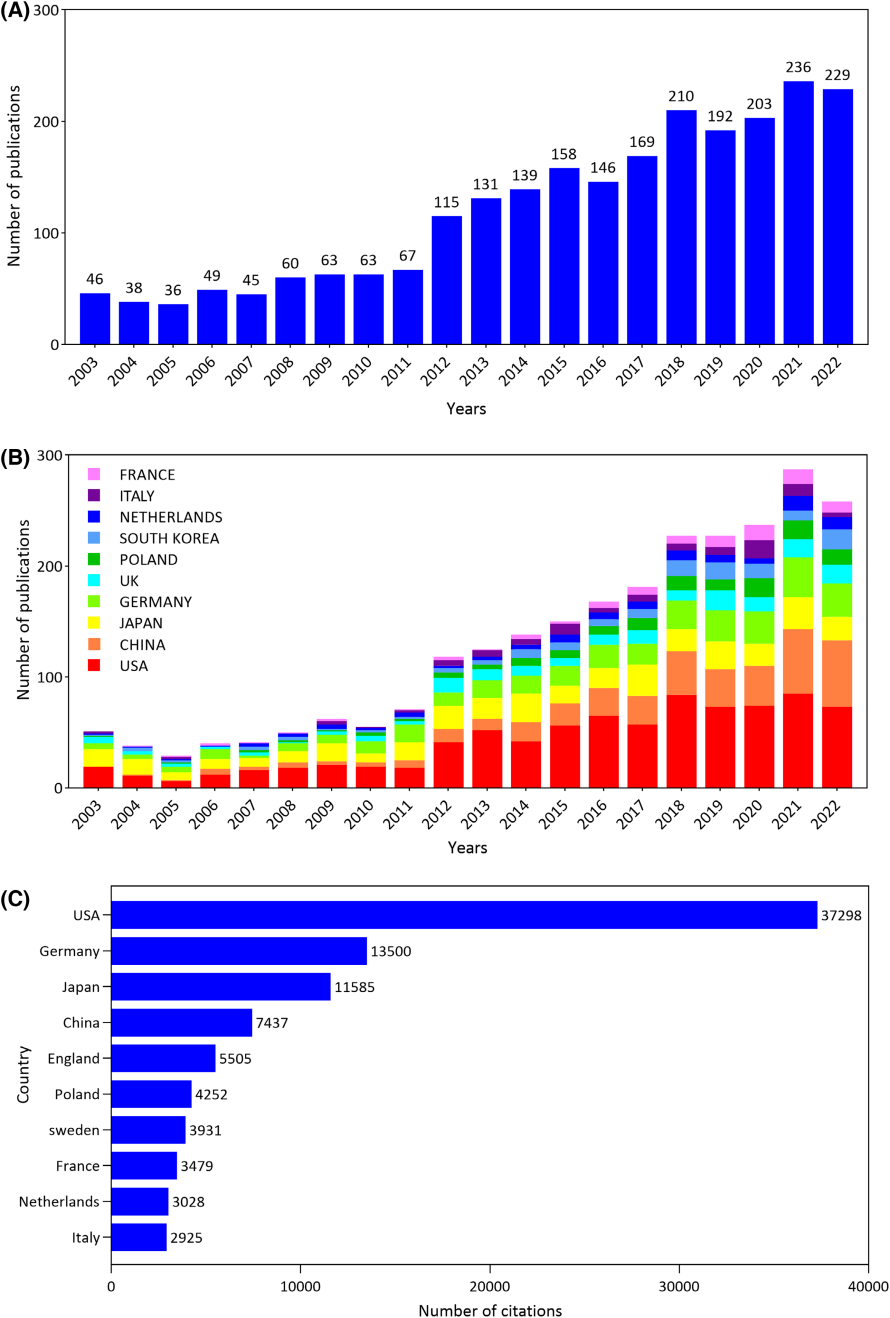
Analysis of the quantity and trends of annual publications. (A) Number and trends of publications on pruritus from 2003 to 2022, export of results from Web of Sciences. (B) Number of publications and growth trends for the top 10 countries/regions in the itch field from 2003 to 2022, export of results from the Bibliometrics online analysis platform. (C) Bar graph of the total number of citations from 2395 retrieved articles among countries in 2003–2022. The top 10 countries with the highest total number of citations are shown. Each bar represents a country, and the length is positively correlated with the total number of citations.

We retrieved 2395 articles from the SCI‐E of WoSCC that met our inclusion criteria. The quantity and trends of publications can reflect the current status of a research field; these are important indicators that help evaluate developments and predict future evolution in the field.^30^ As shown in Figure 2A, the publication volume of papers has mostly shown a continuous increase over the last 20 years. Based on the number of articles published per year, research related to pruritus can be broadly divided into two stages. The initial or early stage (2003–2011) showed a slow growth rate in the number of publications per year. The following decade (2012–2022) was a stage of rapid development in the field. These findings indicate that increasing numbers of scholars are maintaining their interest in the field and conducting related research.

To determine which countries/regions are leading in this field, we analyzed publications in different countries/regions by using the Bibliometrics online analysis platform. The histogram in Figure 2B shows the number of publications from the top 10 countries over the last 20 years. We found a steady increase in the number of pruritus‐related publications in the USA. Although China initially lagged in publication volume, the annual publication outputs have recently grown quickly. The number of citations can reflect the impact of the article in a research field. Figure 2C shows the top 10 countries/regions according to the total number of citations. During this 20‐year period, the total number of citations of articles published in the USA ranked first with 37,298. Germany and Japan ranked second and third with 13,500 and 11,585. Based on the total number of publications and total citations for each country, the average number of citations per article in each country can be derived (total number of citations per country/total number of publications per country). The top 10 countries with the highest total number of citations on itch research during 2003–2022 are listed in Table 1, sorted by the average number of citations. The top three countries with the highest average number of citations per article are Sweden (70.20 citations per article), the USA (44.30) and Germany (42.45). Meanwhile, the country with a relatively lower average number of citations is China (20.32).

**TABLE 1:** The top 10 countries with the highest total number of citations on itch research during 2003–2022 (sorted by the average number of citations).

Rank	Country	Number of publications	Total number of citations	Average number of citations
1	Sweden	56	3931	70.20
2	USA	842	37,298	44.30
3	Germany	318	13,500	42.45
4	France	86	3479	40.45
5	England	142	5505	38.77
6	Poland	125	4252	34.02
7	Japan	347	11,585	33.39
8	Italy	90	2925	32.50
9	Netherlands	94	3028	32.21
10	China	366	7437	20.32

### 3.2 Analysis of collaborating countries/regions and institutions

**FIGURE 3:**
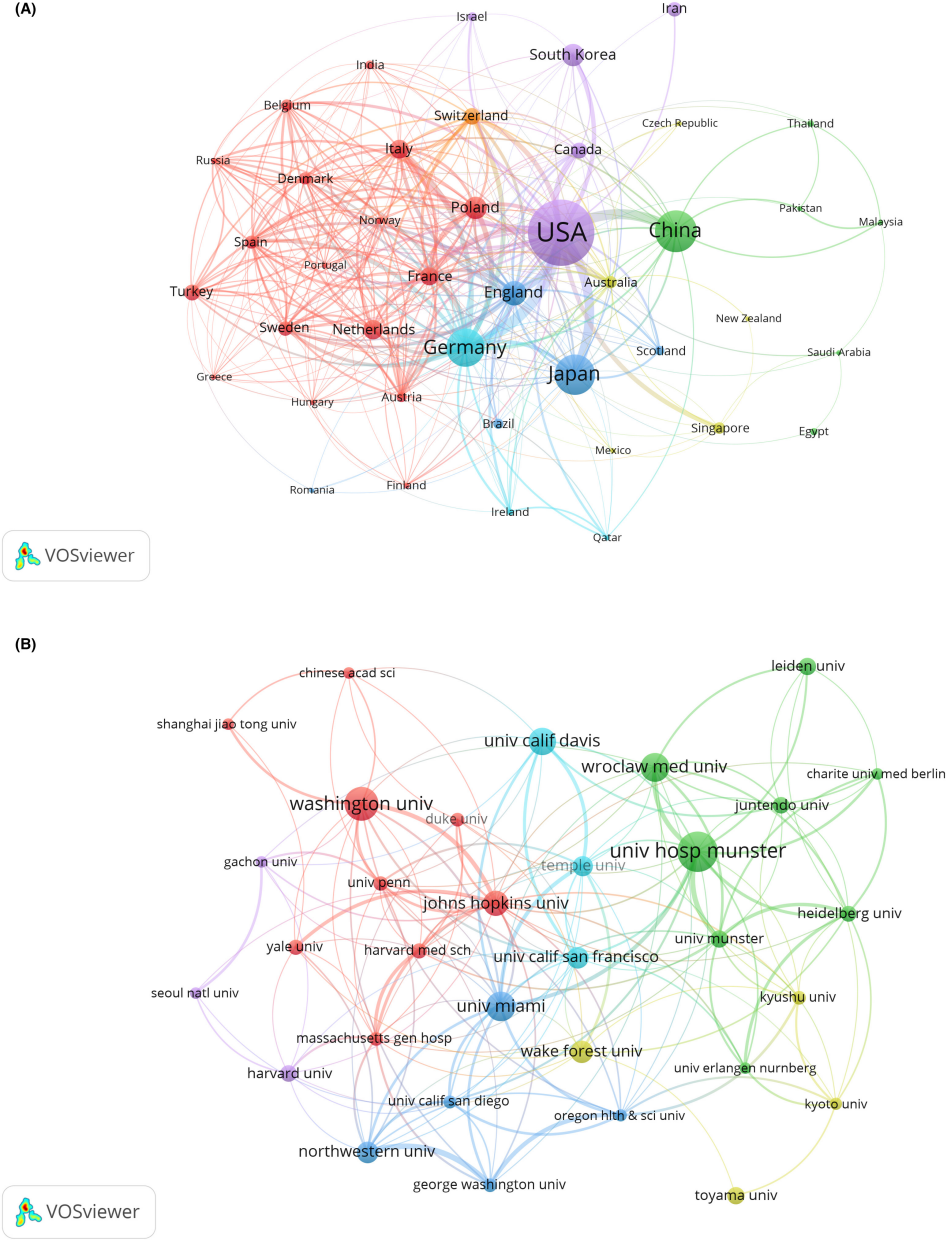
Collaborative network map of countries/regions and institutions. (A) Countries/regions (publications ≥5); (B) Institutions (publications ≥20). The circles represent different countries/regions and institutions, and the size of the circles indicates the number of published articles. The thickness of the links indicates the intensity of cooperation, and the clusters of different colors represent the countries/regions and institutions with strong collaborative relationships.

The 2395 identified articles were published in 72 countries and regions from 2003 to 2022. VOSviewer software was applied to analyze the academic collaborations between the countries/regions. As shown in Figure 3A, countries with ≥5 publications are displayed. The circles represent different countries/regions, and the size of the circles indicates the number of articles published in each country/region. The thickness of the links indicates the degree of cooperation between countries, and the different colors represent the clusters of cooperating countries. The highest total number of documents was published by researchers from the USA, followed by Japan and Germany. We found that articles on itch were mainly published in Europe, North America, and Asia. Most countries in the red cluster were in Europe and showed greater collaborations compared to other clusters. The USA was centrally located in international cooperative efforts. The USA and Germany were the most frequently collaborating countries.

To clarify the interinstitutional collaborations in the field, we used VOSviewer to build a visual mapping of institutions. 2646 institutions contributed to pruritus research; Figure 3B lists the productive institutions with ≥20 publications. The visualization results showed that within‐country/region collaborative networks were particularly intensive. University Hospital Münster in Germany published the most articles and had stronger connections to other institutions. Table S1 lists the top 10 most productive institutions. 8 of the top 10 most productive institutions were from the USA, indicating that American institutions play a key role in the itch research field.

### 3.3 Analysis of co‐authorship networks and core author distribution

**FIGURE 4:**
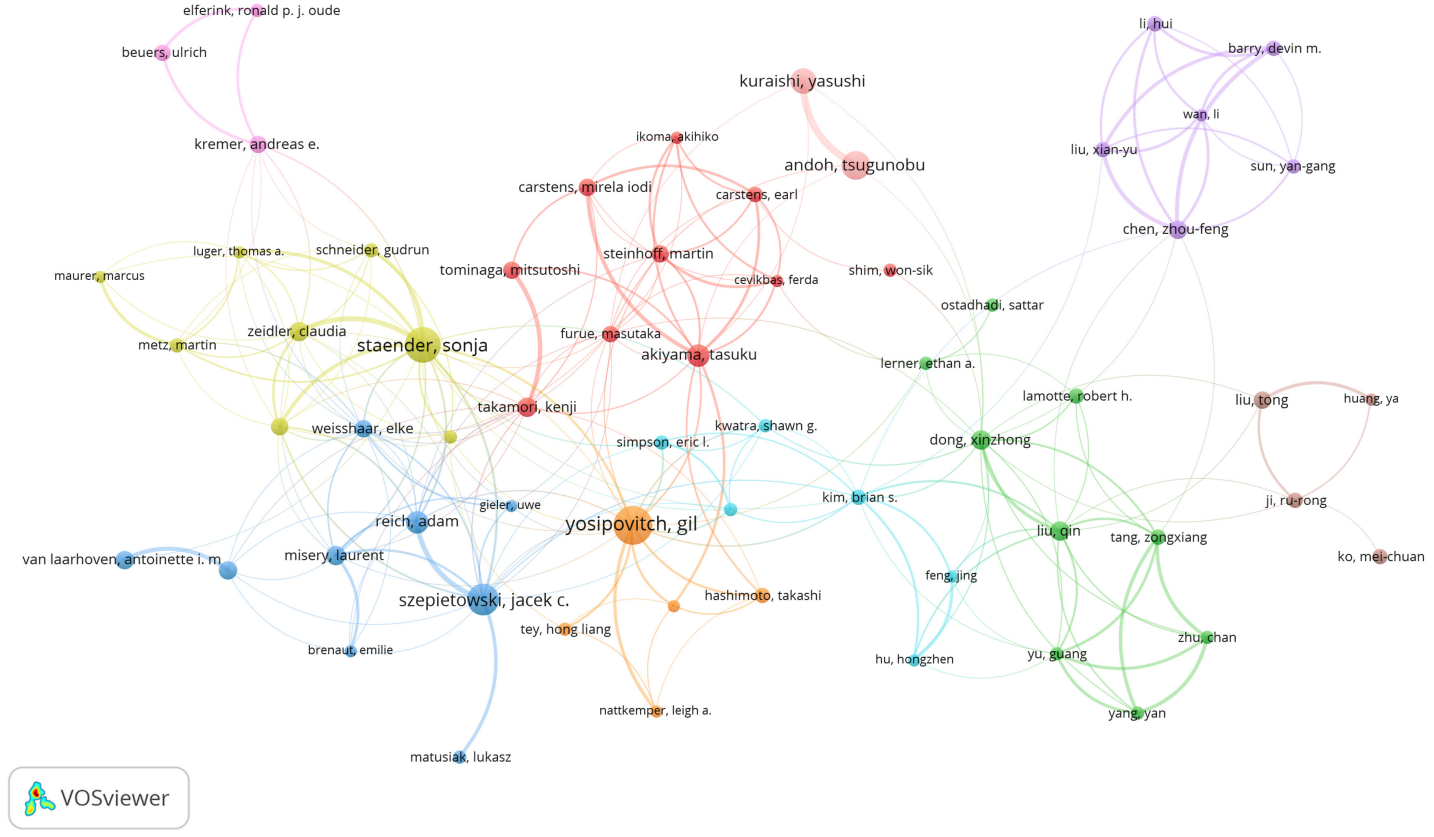
VOSviewer network of authorship in the itch research field. The 63 authors with the highest number of publications (number ≥10) are shown. Each circle represents an author and links between two circles indicate cooperation with each other. Font size is positively correlated with the quantity of published papers.

Study completion often requires the collaboration of multiple researchers. By analyzing the features of the author collaboration network, we can identify the core authors of a given research field and the extent of inter‐author collaborations.^31^ Over the past 20 years, 11,365 authors contributed to the 2395 identified articles. The visualization of author collaborations is shown in Figure 4. Here, we labeled the top 63 authors, defined as researchers who published ≥10 papers. The node and font size are positively correlated with the number of published articles, and the thickness of the linking lines represents the density of collaboration between authors. Table S2 lists the countries and the total citations of these authors. The most published author in this field was Gil Yosipovitch from the Itch Research Center at the University of Miami, USA, with 88 publications and 3169 citations. His research team has been focusing on neuroimmune mechanisms and clinical treatment in chronic pruritus.^32^ This was followed by Sonja Staender (84 papers) and Jacek C Szepietowski (50 papers). Most of these prolific authors were from highly productive countries or institutions and showed strong collaborative relationships.

### 3.4 Analysis of journals

The 2395 included publications came from 638 different journals. The influence of the journals was analyzed using the bibliometric online analysis platform, and the top 10 journals according to the number of total citations are shown in Table 2. *Acta Dermato‐Venereologica* had the highest number of publications and citations, with 148 articles and 1401 citations. This was followed by the *British Journal of Dermatology* (856), *Journal of Neuroscience* (758), *Pain* (606), *Nature Neuroscience* (497), *Neuron* (484), *Journal of Allergy and Clinical Immunology* (480), *Journal of the American Academy of Dermatology* (455), *Cell* (444), and *Science* (396). Eight of these journals were from the United States of America. Articles published in *Cell* had the highest number of average citations per article (88.8), followed by *Science* (79.2), and *Nature Neuroscience* (71).

**TABLE 2:** The top 10 most cited journals in the itch field (sorted by total citations).

Rank	Journal title	Frequency	Total citations	Average citation per paper	Impact factor (2022)	JCR	Country
1	*Acta Dermato‐Venereologica*	148	1401	9.47	3.6	Q1	Norway
2	*British Journal of Dermatology*	65	856	13.17	10.3	Q1	England
3	*Journal of Neuroscience*	34	758	22.29	5.3	Q1	USA
4	*Pain*	34	606	17.82	7.4	Q1	USA
5	*Nature Neuroscience*	7	497	71	25.0	Q1	USA
6	*Neuron*	16	484	30.25	16.2	Q1	USA
7	*Journal of Allergy and Clinical Immunology*	23	480	20.87	14.2	Q1	USA
8	*Journal of the American Academy of Dermatology*	52	455	8.75	13.8	Q1	USA
9	*Cell*	5	444	88.8	64.5	Q1	USA
10	*Science*	5	396	79.2	56.9	Q1	USA

### 3.5 Analysis of co‐citation and clustered network

**FIGURE 5:**
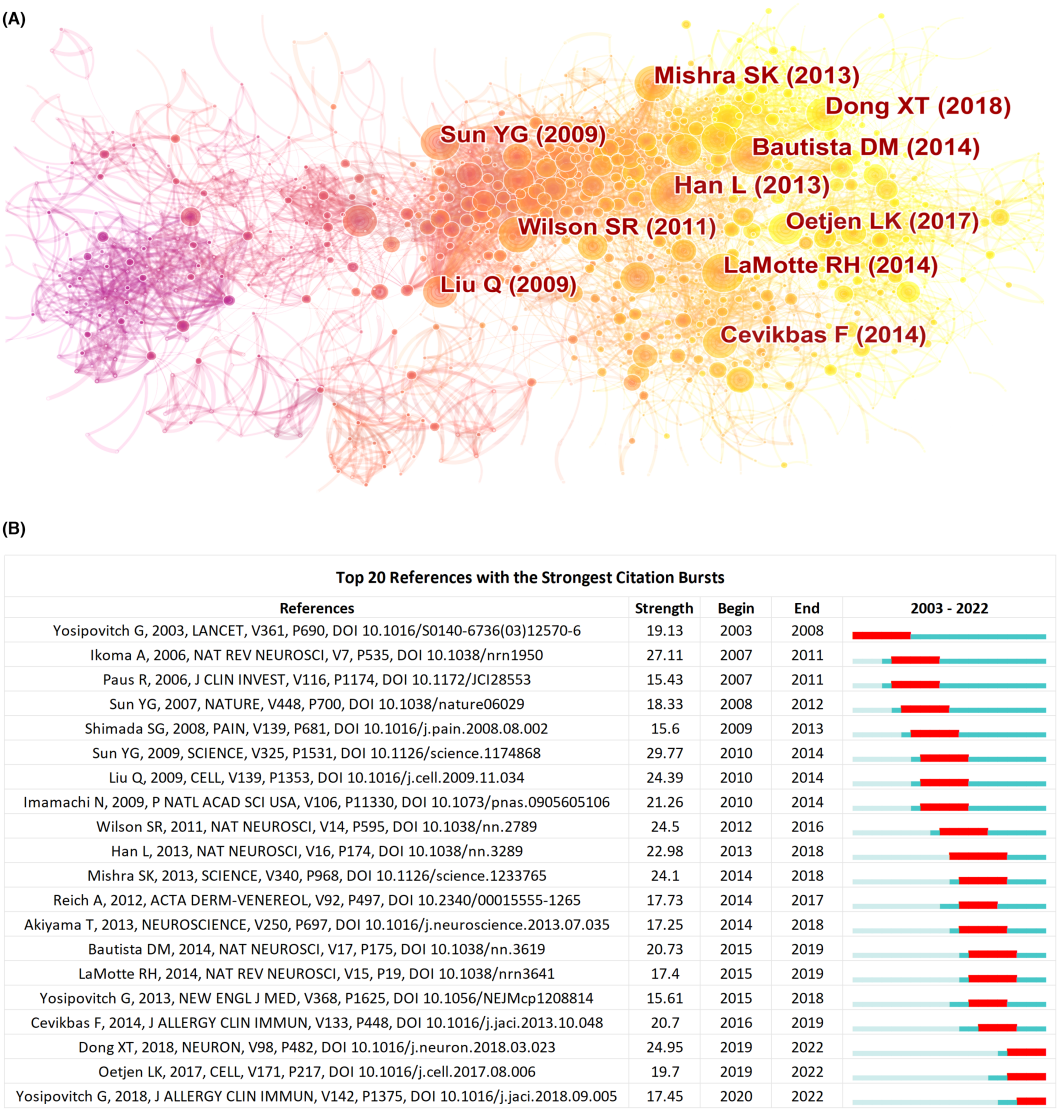
Reference co‐citation network analysis of publications in the field of itch between 2003 and 2022. (A) CiteSpace co‐citation map of 43,341 references on itch research. Each node represents a reference, the size of the node is positively correlated with the citation frequency, and the link between two nodes represents two references cited in the same article. Yellower nodes represent papers that have been cited frequently in recent years, while redder nodes represent references that were cited in earlier years. The published year and first author of the top 10 most cited publications are shown. (B) The top 20 references with the strongest citation bursts for articles on itch research from 2003 to 2022. The blue bars indicate the period in which the reference has been published, and the red bars represent bursts of citation frequency.

To some extent, citations can serve as a proxy for article content. Based on this, co‐citation analysis is an effective way to estimate the relevance of two studies. The 2395 publications and 43,341 references were analyzed by using CiteSpace software to determine the homogeneity of the highly cited literature in the itch research field and to conduct a cluster analysis. Figure 5A identifies the top 10 references sorted by co‐citation frequency. Each node represents one reference, and the node size is positively correlated with the citation frequency. The links between nodes indicate that these articles were cited as references in the same articles, and the thickness of the lines indicates the relevance between the cited papers. The different colors of the nodes reveal different citation periods, with red indicating papers cited in earlier years and yellow indicating papers frequently cited more recently. Table 3 lists the top 10 references sorted by frequency of co‐citation. The results revealed that the most cited literature was a basic study published in *Nature Neuroscience* in 2013.^33^ This study identified MrgprA3^+^ neurons as a specific subpopulation that mediates itch in the DRG of mice after the induction of a dry skin inflammation model or AD model. The second‐ranked article was published in *Nature Neuroscience* in 2014.^34^ This paper discussed the role of neurons, keratinocytes, and inflammatory cells in acute and chronic pruritus, assessing the role of signaling molecules released by inflammatory cells, such as mast cells and basophils, to mediate itch. The third article was published in *Science* in 2013.^7^ This study characterized the neuropeptide natriuretic peptide b (Nppb) as an itch‐associated neuropeptide, demonstrating that Nppb was expressed in a subpopulation of TRPV1^+^ neurons. These three most highly cited articles explored the specific mechanisms mediating itch signals from a molecular perspective and provided a powerful theoretical basis for the development of novel therapies to alleviate chronic itch, including inflammatory pruritus. As such, these highly cited references have greatly contributed to itch research.

**TABLE 3:** The top 10 high‐cited references of 2395 retrieved articles on itch research from 2003 to 2022 (sorted by cited frequency).

Rank	Title	First Author	Source	Year	Cited frequency	DOI
1	A subpopulation of nociceptors specifically linked to itch	Liang Han	*Nat Neurosci*	2013	82	https://doi.org/10.1038/nn.3289
2	Why we scratch an itch: the molecules, cells, and circuits of itch	Diana M Bautista	*Nat Neurosci*	2014	77	https://doi.org/10.1038/nn.3619
3	The cells and circuitry for itch responses in mice	Santosh K Mishra	*Science*	2013	71	https://doi.org/10.1126/science.1233765
4	Peripheral and Central Mechanisms of Itch	Xintong Dong	*Neuron*	2018	70	https://doi.org/10.1016/j.neuron.2018.03.023
5	Cellular basis of itch sensation	Yan‐Gang Sun	*Science*	2009	67	https://doi.org/10.1126/science.1174868
6	Sensory neurons and circuits mediating itch	Robert H LaMotte	*Nat Rev Neurosci*	2014	65	https://doi.org/10.1038/nrn3641
7	TRPA1 is required for histamine‐independent, Mas‐related G protein‐coupled receptor‐mediated itch	Sarah R Wilson	*Nat Neurosci*	2011	64	https://doi.org/10.1038/nn.2789
8	Sensory Neurons Co‐opt Classical Immune Signaling Pathways to Mediate Chronic Itch	Landon K Oetjen	*Cell*	2017	61	https://doi.org/10.1016/j.cell.2017.08.006
9	A sensory neuron‐expressed IL‐31 receptor mediates T helper cell‐dependent itch: Involvement of TRPV1 and TRPA1	Ferda Cevikbas	*J Allergy Clin Immun*	2014	61	https://doi.org/10.1016/j.jaci.2013.10.048
10	Sensory neuron‐specific GPCR Mrgprs are itch receptors mediating chloroquine‐induced pruritus	Qin Liu	*Cell*	2009	55	https://doi.org/10.1016/j.cell.2009.11.034

The references burst refers to papers showing a rapid increase in citation frequency, which usually represents the emergence or transformation of a research field. In addition, the higher burst strength of the reference shows greater significance in the field.^35^ As shown in Figure 5B, the top 20 references with the strongest citation bursts for the past 20 years were generated using CiteSpace. The blue line indicates the time frame from 2003 to 2022, and the red line indicates the period over which the burst references were maintained. Among the burst references in recent years, the latest was a review published in the *Journal of Allergy and Clinical Immunology* in 2018.^36^ The burst occurred from 2020 until the end of 2022. This review provided an overview of the underlying molecular, neural, and immune mechanisms of itch as well as novel therapeutic approaches. The second reference was an article published in *Cell* in 2017 and the burst occurred from 2019 until the end of 2022.^18^ This study explored the mechanisms of sensory neurons and immune signaling pathways mediating chronic itch and also showed that JAK inhibitors could significantly alleviate pruritus in patients with persistent chronic pruritus who had failed other immunotherapy. The third reference was a review published in *Neuron* in 2018, which burst from 2019 until the end of 2022. This review summarized the understanding of the molecular and neural mechanisms of itch and discussed the neural circuits involved in itch processing in the peripheral and central nervous systems.^11^ Among these 20 references, the basic research paper with the highest strength was published in *Science* in 2009 and this burst occurred from 2010 until 2014.^4^ In this research, the cellular basis of itch sensation was explored and the GRPR was, for the first time, identified as a pruritus‐related specific receptor.

**FIGURE 6:**
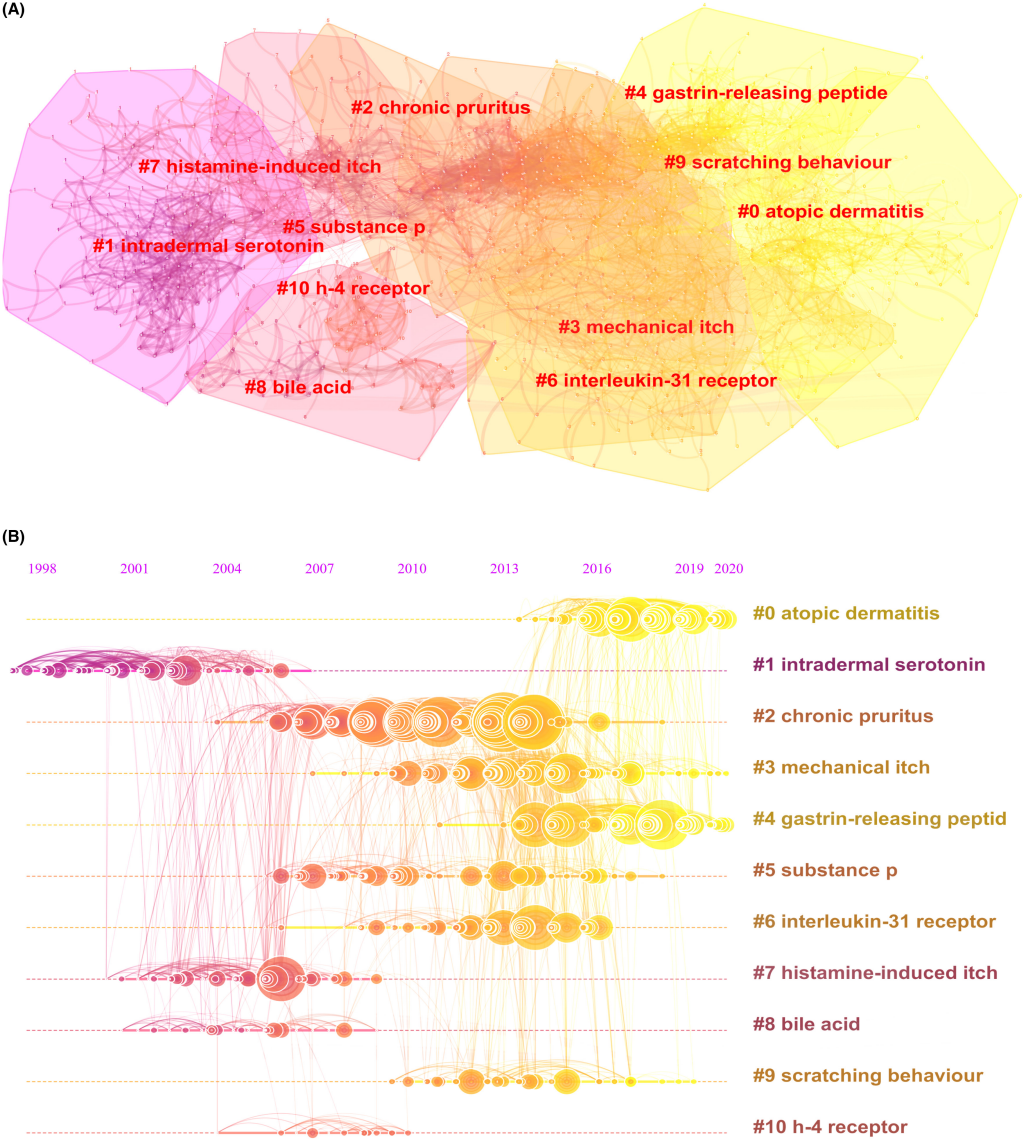
Analysis of Clustered Network of publications on itch research between 2003 and 2022. (A) CiteSpace visual analysis of the clustering network of co‐cited literature. The top 11 largest citation clusters are shown. (B) Timeline view of co‐cited references related to itch. Each horizontal line represents a cluster, and the smaller the label number is, the larger the cluster. The node size reflects the co‐citation frequency and the links indicate co‐citation relationships. The colors of nodes and lines represent the different years cited.

CiteSpace was used to conduct a co‐citation cluster mapping of the references in the 2395 articles based on keywords. As shown in Figure 6A, the number of cluster labels was negatively correlated with the number of articles included in each cluster. The more yellow clusters indicate that the references in these clusters were cited more frequently in recent years. The co‐citation cluster analysis showed the most popular terms in the field of itch research by hierarchical cluster labels, including 0# atopic dermatitis, 1# intradermal serotonin, 2# chronic pruritus, 3# mechanical itch, 4# gastrin‐releasing peptide, 5# substance p, 6# interleukin‐31 receptor, 7# histamine‐induced itch, 8# bile acid, 9# scratching behavior, and 10# h‐4 receptor. A summary of the clusters is presented in Table 4. The silhouette value of >0.5 indicates that the clustering results were credible.^24^

**TABLE 4:** A summary of 11 clusters of the references in publications of itch.

Cluster ID	Term	Size	Silhouette^a^
0	Atopic dermatitis	164	0.848
1	Intradermal serotonin	160	0.94
2	Chronic pruritus	158	0.866
3	Mechanical itch	122	0.86
4	Gastrin‐releasing peptide	101	0.9
5	Substance p	75	0.871
6	Interleukin‐31 receptor	71	0.864
7	Histamine‐induced itch	65	0.965
8	Bile acid	47	0.937
9	Scratching behavior	38	0.934
10	h‐4 receptor	19	0.992

^a^ The silhouette value of >0.5 indicates that the clustering results are credible.

### 3.6 Analysis of research trends and burst detection

To illustrate the changes in itch research hotspots over the past 20 years, Figure 6B shows a timeline view of the cited publications. As shown, each circle represents the main cited literature in the cluster, while the size of the circles on the timeline indicates the frequency of citations. Intradermal serotonin was an early research hotspot that lasted from 1997 to 2006. The main research hotspots in recent years were chronic pruritus, substance p, and interleukin‐31 receptor; this started in approximately 2006 and ended in 2016. However, the latest hotspots included AD and GRP, which emerged in 2013 and continue to this day. The involvement of immunity and inflammation in the development of itch has been a major research hotspot in the field.

**FIGURE 7:**
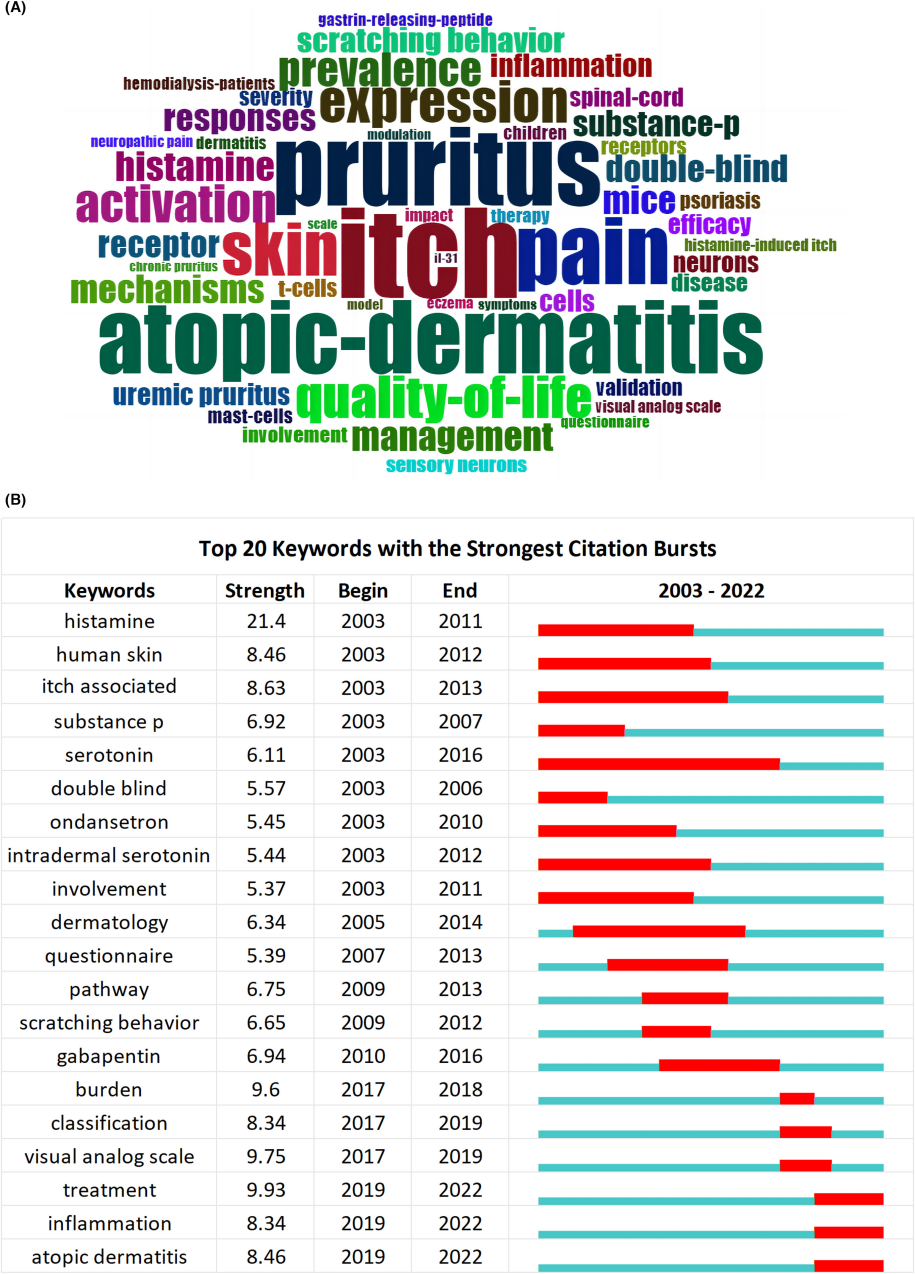
Analysis of keywords and burst detection for articles related to itch from 2003 to 2022. (A) Word cloud analysis of the top 50 high‐frequency keywords on itch. The font size represents the occurrence frequency of keywords. (B) The top 20 keywords with the strongest citation bursts (sorted by beginning year). Keywords marked with red bars indicate a sudden increased frequency of use of the keyword during that period, and blue represents a relatively unpopular time period.

A word cloud was generated for the top 50 high‐frequency keywords with the R package “Bibliometrix,” as shown in Figure 7A. Among these high‐frequency keywords, in addition to the search terms “itch” and “pruritus,” other high‐frequency keywords included “atopic dermatitis,” “expression,” “quality of life,” “activation,” “histamine,” “management,” “substance p,” “inflammation,” “t cells,” “mast cells,” and “therapy”. This suggested that neuroimmunology and inflammation, as well as treatment or management, are currently emphasized in itch research.

Keywords burst analysis is a valuable approach to identifying the trend of the research field over a specific period and can help researchers quickly capture research hotspots.^37^ Figure 7B shows the top 20 keywords with the strongest bursts in the itch field over the past 20 years. The blue line indicates the time range of 2003–2022, and the red line indicates a burst period in keyword occurrence. Among these keywords, the keyword with the strongest burst was “histamine” (21.4). Histamine is a classic inflammatory mediator released by mast cells that activates histamine receptors in peripheral nerves, causing the release of neuropeptides that aggravate inflammatory responses in the skin.^38^ The second‐ranked keyword was “treatment,” with a strength of 9.93 and the third keyword was the “visual analog scale” (9.75). The diagnosis and treatment of pruritic diseases were also hotspots in clinical studies. The keywords “inflammation” and “AD” also burst until 2022, with strengths of 8.34 and 8.46, respectively. Both were strongly associated with itch and have become current research hotspots

## 4 DISCUSSION

This study is the first bibliometric and visual analysis of research on pruritus. We retrieved 2395 publications that were closely related to the study of itch, published between 2003 and 2022, from the SCI‐E of WOSCC. The collaboration network analysis showed that the USA (country), University Hospital Münster (institute), and Gil Yosipovitch (investigator) were the most influential in the field. Keyword burst detection indicated that treatment, inflammation and AD are the current research hotspots. With the help of this bibliometric analysis, researchers interested in this field can rapidly gain a grasp of the current research hotspots.

The annual volume and trend of published articles can reflect the rate of development and research progress in a given field. The scientific output dedicated to itch has generally maintained a steady growth trend over the last two decades. The relatively low volume of publications from 2003 to 2011 showed that to be an early developmental period in itch research. Since 2012, the number of publications per year fluctuated slightly, but there was an overall trend of vigorous growth, indicating an increasing interest in itch.

Collaborative network analysis enabled the assessment of cooperative relationships between countries, institutions, and scholars. Over the past 20 years, the USA was at the center of international cooperative partnerships with other highly productive countries. Seven of the top 10 institutions, in terms of the number of published articles, were from the USA. Sweden ranked at the top in terms of the average citations, followed by the USA and Germany, demonstrating the high quality of publications in these countries. The University Hospital Münster of Germany published the largest number of articles, indicating that it has made an important contribution to the itch field. Continuous improvements in communication and collaboration between countries and institutions could facilitate driving this research field forward even further.

Among the authors who have contributed to the field of pruritus over the past 20 years, Gil Yosipovitch, Sonja Staender, and Jacek C Szepietowski were the three most productive authors. Gil Yosipovitch is a tenured professor in the Department of Dermatology at the University of Miami, USA, and director of the Miami Pruritus Center, which focuses on chronic pruritus. Prof. Yosipovitch's group has been focusing on the neuroimmune mechanisms and clinical treatment of pruritus.^36^ Professor Sonja Staender from University Hospital Münster, Germany, mainly focuses on the pharmacological treatment of inflammatory pruritic diseases such as AD.^39^ Professor Jacek C Szepietowski from Wroclaw Medical University, Poland, is mainly interested in the diagnosis and evaluation of pruritic diseases including psoriasis.^40^, ^41^

The analysis of source journals can assist researchers to identify the core journals in their research fields. Among the 10 most frequently cited journals in the field, *Acta Dermato‐Venereologica* published the most articles and also had the most citations, followed by the *British Journal of Dermatology*, and the *Journal of Neuroscience*. Articles published in Cell had the highest average citations per article, followed closely by *Science*, both of which are globally recognized and authoritative journals. The scientific directions covered by these journals focused on neuroscience, immunology, dermatology, and integrative fields, indicating that the current research themes in itch are mainly focused on neuroimmunology.

Co‐cited references can offer significant information on the frontiers of scientific research.^42^ The top 10 most frequently cited papers reported on studies into the molecular mechanisms of itch, including neural and immunological circuits, as well as mediators of itch such as GRPR, neuropeptide natriuretic peptide b (Nppb), and IL‐31. The most‐cited article was a study published by Han et al. in *Nature Neuroscience* in 2013.^33^ This study defined and characterized a specific subpopulation of itch‐mediated neurons, opening a new avenue for the investigation of pruritus and the development of antipruritic therapies.

Reference bursts usually indicate the emergence or transformation of a research field. Among the top 20 references with the strongest citation bursts over the past 20 years, two of the latest among the burst citations in recent years were two reviews published in 2018.^11^, ^36^ These two reviews provided a detailed summary of advances in the study of the molecular mechanisms of itch and proposed innovative approaches to itch therapy. These have contributed to providing direct and insightful guidance for itch research in recent years. Another one among the burst citations in recent years was an article published in *Cell* in 2017.^18^ This study demonstrated that type 2 cytokines can directly activate sensory neurons in mice and humans in a JAK–STAT signaling pathway‐dependent manner. The discovery of neuroimmune involvement in itch provided new insights into its mechanisms and clinical treatment approaches

Co‐citation cluster analysis can be applied to identify major research themes. As shown in Figure 6B, “intradermal serotonin,” “histamine‐induced itch,” and “h‐4 receptor” were the main research themes in the early stage of research publication history. During inflammatory disease, mast cells can be activated by various factors, among which immunoglobulin E (IgE) is one of the classical pathways that trigger itch. IgE attaches to the cell membrane via the high‐affinity receptor FcRI and rapidly induces mast cell degranulation to release various pruritic molecules, including histamine and serotonin.^43^, ^44^ The main research themes during the last decade have surrounded the neural and itch‐specific molecules or pathways related to immunity, including “GRP,” “interleukin‐31 receptor,” and “substance p.” The exploration of the mechanisms of itch production in “AD,” a pruritic disease closely related to neuroimmunity, has been of great research interest in recent years.

Keyword bursts refer to keywords that are cited extensively in publications and may help to identify trends and hotspots in a research field.^29^ Figure 7B shows the top 20 keywords with the strongest bursts in the itch field over the past 20 years. The strongest keyword was “histamine,” indicating that histamine research was a significant early hotspot, which matched the co‐citation cluster analysis. Histamine was first studied 112 years ago^45^ and is one of the most important pruritic mediators. Previously, researchers focused on the typical IgE‐mast cell‐histamine axis to investigate the mechanisms of itch.^46^, ^47^ The second keyword was “treatment”. Histamine and its receptors have been frequently identified as targets for chronic pruritic diseases in previous studies.^48^ However, the efficacy of antihistamines is limited and their long‐term use can lead to the aggravation of skin conditions such as AD.^49^ Remarkable advances have been achieved in pruritus treatment, and future studies should determine which of these therapies offers the strongest and most specific efficacy for clinical pruritus.

The third strongest keyword was “visual analog scale.” Itch is a subjective symptom with a multidimensional nature and assessment of pruritus is necessary to standardize treatment and care. The visual analog scale (VAS) is currently the most commonly used approach to assess pruritus. Initially, the VAS was used to assess pain but later studies confirmed that the VAS is also applicable to pruritus assessment.^50^ The VAS is easy to use valid reliable and reproducible. However, the response is unidimensional can be influenced by the individual's cognition and understanding and requires physician guidance.^51^ Therefore, other tools that assess the impact of itch intensity on quality of life and patient satisfaction should also be integrated into routine treatment and care.^52^ Considerable work is required to develop valid and sensitive universal itch assessment scales and objective measures

The keywords “inflammation” and “AD” are also current research hotspots; these started to burst out in 2019 and have continued until the end of 2022. Itch and inflammation are closely related, and itch sensation often occurs in the context of neuroinflammation. In addition to inflammatory factors affecting itch, multiple neuropeptides released by sensory nerves such as CGRP, SP, and VIP have various itch‐modulatory mechanisms while also facilitating skin inflammation.^21^, ^22^, ^23^ These neuropeptides can interact with a variety of immune cells, including eosinophils, mast cells, macrophages, and T cells, to induce inflammation.^53^ Thus, the role of inflammation in regulating itch sensation is complex and may require more mechanistic understanding; this may drive future research targets. AD is a chronic inflammatory skin disease characterized by recurrent episodes of intense pruritus.^54^ The itch‐scratch cycle can further aggravate the inflammatory skin response, resulting in uncontrollable pruritus.^55^ In recent years, biological agents and small molecule compounds have been used to benefit some AD patients, as the involved neuroimmune circuits have been studied more intensively.^56^ However, there are many remaining unknowns, and the discovery of new therapeutic targets is expected to provide more precise and effective treatment options for AD and other inflammatory skin diseases.

Overall, although itch has been scientifically recognized for 360 years, we have only begun to gradually understand its underlying mechanisms in the last 20 years. Itch and neuroimmunology research has identified potential mechanisms in terms of nerve transmission,^57^ immune cells,^15^ and inflammatory factors,^16^ which provide the basis for biologically targeted agents to treat pruritic diseases. However, various research directions still deserve further exploration and focus. First, neuroimmunology will remain a major research hotspot in future studies of itch mechanisms. In particular, it is essential to explore which immune pathways specifically modulate itch sensation and whether they are associated with the activation of certain neurons. Second, as there are no suitable drugs specifically for pruritus treatment in clinical practice, and an increasing number of studies have indicated that type 2 immunity and pruritus are closely related,^58^ it is necessary to identify new targets for pruritus treatment aimed at these immune‐related cells and cytokines. Next, increasingly, research studies have suggested that microglia and astrocytes critically mediate nervous system inflammation.^59^ The neuroinflammation of pruritic skin diseases is further exacerbated by neuroimmune modulation of the itch‐scratch cycle.^8^ Thus, research on the relationship between neuroinflammation and itch sensation will provide new understanding and insight into alleviating scratching behavior in pruritic diseases. Finally, pruritus is a chronic syndrome, especially in patients who have failed long‐term treatment, and is often accompanied by psychosomatic comorbidities such as anxiety and depression.^60^ Therefore, clinical diagnosis and assessment of pruritus should be accompanied by the evaluation of the patient's psychiatric and psychological status. Moreover, exploring the neurological and immunological mechanisms by which pruritus affects the patient's psychiatric status may be a future research direction.

This study has some limitations. First, due to the limitation of the CiteSpace software, only data extracted from SCI‐E of the WoSCC database could be analyzed visually. Records from other important search engines, such as PubMed, Embase, and other databases were excluded. Although WoSCC is one of the best‐known and high‐quality databases,^61^ its exclusive use may lead to some bias. Second, due to the search strategy, type of literature, and language restrictions, our study focused only on papers published in English; English is still the preferred language of academic journals today. This may have led us to miss articles that did not meet these criteria. Finally, bibliometric analysis has a potential time effect bias. Older papers may often receive more citations, and many other factors influence citation rates, such as self‐citation.^62^ We attempted to reduce the risk of such errors by conducting a rigorous review of included articles.

## 5 CONCLUSIONS

In conclusion, this is the first bibliometric and visual analysis of itch research. Over the last 20 years, the number of publications in the itch research field has increased steadily. In the last decade, the number of publications has increased rapidly, indicating a growing interest by researchers in the field. The USA was the most active country in international scientific collaborations. In addition, the current research theme is focused on inflammation and neuroimmunology. Therefore, exploring the neuroimmunological and inflammatory mechanisms of itch, as well as improvements and innovations in clinical assessment or therapeutic targets for itch will be very significant research directions in the future. We hope that this bibliometric analysis will provide useful information for future itch research.

## AUTHOR CONTRIBUTIONS

Po Gao, Liqun Yang, and Xiaoqiong Xia raised the conception of the study and designed the study. Jun Li, Liya Wang, and Suqing Yin conducted the CiteSpace and VOSviewer analysis. Jun Li, Po Gao, and Liya Wang screened articles and wrote the original manuscript. Po Gao, Liqun Yang, Xiaoqiong Xia, Jun Li, Liya Wang, Suqing Yin, Shuangshuang Yu, Yanyu Zhou, Xiaoqi Lin, Yingfu Jiao, and Weifeng Yu revised the manuscript and edited critically. All authors contributed to the article and approved the submitted version.

## CONFLICT OF INTEREST STATEMENT

The authors declare no competing interests.

## Supporting information


Table S1.
Table S2.

## Data Availability

The data that support the findings of this study are available from the corresponding author upon reasonable request.
