# Supplementary material for: Emerging trends and hotspots of the itch research: A bibliometric and visualized analysis
Source: CNS Neurosci Ther. 2023 Oct 30;30(4):e14514. doi: 10.1111/cns.14514 (PMC11017449; doi:10.1111/cns.14514)
Supplement: Supplementary file 1 — Table S1. Table S2. [file CNS-30-e14514-s001.docx]

Supplementary Material

Emerging Trends and Hotspots of the Itch Research: A Bibliometric and Visualized Analysis

Jun Li^1, 2,^ ^3^, Liya Wang^4^, Suqing Yin ^1, 2^, Shuangshuang Yu^5^, Yanyu Zhou^1, 2^, Xiaoqi Lin^1, 2^, Yingfu Jiao^1, 2^, Weifeng Yu^1, 2^, Xiaoqiong Xia^3, *^, Liqun Yang^1, 2, *^, Po Gao^1, 2, *^

^1^Department of Anesthesiology, Renji Hospital, Shanghai Jiao Tong University School of Medicine, Shanghai 200127, China

^2^Key Laboratory of Anesthesiology (Shanghai Jiao Tong University), Ministry of Education, China

^3^Department of Anesthesiology, Chaohu Hospital Affiliated to Anhui Medical University, Chaohu 238000, Anhui, China

^4^Department of Gynecologic Oncology, International Peace Maternity and Child Health Hospital, Shanghai Jiao Tong University School of Medicine, Shanghai Municipal Key Clinical Specialty, Shanghai Key Laboratory of Embryo Original Disease, Shanghai 200030, China

^5^Department of Dermatology, Chaohu Hospital Affiliated to Anhui Medical University, Chaohu 238000, Anhui, China

*** Correspondence**

Po Gao and Liqun Yang, Department of Anesthesiology, Renji Hospital, Shanghai Jiao Tong University School of Medicine, Shanghai, China.

Email: [gaopo0908@163.com](mailto:gaopo0908@163.com) and [lqyang72721@126.com](mailto:lqyang72721@126.com)

Xiaoqiong Xia, Department of Anesthesiology, Chaohu Hospital Affiliated to Anhui Medical University, Chaohu, Anhui, 238000, China.

Email: xxq2366833@sina.com

The first three authors contributed equally to this work.

**Supplementary Table 1.** The top 10 institutions by publication frequency.

| **Rank** | **Institution** | **Country** | **Frequency** | **Percentage** |
| --- | --- | --- | --- | --- |
| 1 | University Hospital Münster | Germany | 80 | 3.34% |
| 2 | University of Washington | USA | 66 | 2.76% |
| 3 | University of Miami | USA | 57 | 2.38% |
| 4 | Medical University of Wroclaw | Poland | 56 | 2.34% |
| 5 | University of California, Davis | USA | 53 | 2.21% |
| 6 | Johns Hopkins University | USA | 48 | 2.00% |
| 7 | Wake Forest University | USA | 42 | 1.75% |
| 8 | Northwestern University | USA | 40 | 1.67% |
| 9 | University of California, San Francisco | USA | 40 | 1.67% |
| 10 | Temple University | USA | 38 | 1.59% |

**Supplementary Table 2.** The top 10 authors with the most publications.

| **Rank** | **Author** | **Publications** | **Citations** | **Country** | **Institution** |
| --- | --- | --- | --- | --- | --- |
| 1 | Gil Yosipovitch | 88 | 3169 | USA | University of Miami |
| 2 | Sonja Staender | 84 | 3031 | Germany | University Hospital Münster |
| 3 | Jacek C Szepietowski | 50 | 2167 | Poland | Wroclaw Medical University |
| 4 | Tsugunobu Andoh | 40 | 869 | Japan | University of Toyama |
| 5 | Yasushi Kuraishi | 35 | 766 | Japan | University of Toyama |
| 6 | Adam Reich | 27 | 1501 | Poland | Medical College of Rzeszow University |
| 7 | Tasuku Akiyama | 27 | 1549 | USA | University of Miami |
| 8 | Qin Liu | 22 | 2156 | USA | University of Washington |
| 9 | Kenji Takamori | 22 | 1120 | Japan | Juntendo University Urayasu Hospital |
| 10 | Claudia Zeidler | 22 | 371 | Germany | University Hospital Münster |
